# Stability assessment of selected chrysanthemum (*Dendranthema grandiflora* Tzvelev) hybrids over the years through AMMI and GGE biplot in the mid hills of North-Western Himalayas

**DOI:** 10.1038/s41598-024-61994-4

**Published:** 2024-06-19

**Authors:** Shilpa Kamal, Amit Rana, Rajni Devi, Ravi Kumar, Niketa Yadav, Aniket Anant Chaudhari, Shimran Yadav, Sanatsujat Singh, Bhavya Bhargava, Satbeer Singh, Ramesh Chauhan, Ashok Kumar

**Affiliations:** 1 Agrotechnology Division, Council of Scientific and Industrial Research - Institute of Himalayan Bioresource Technology, Palampur, Himachal Pradesh 176061 India; 2 Academy of Scientific and Innovative Research (AcSIR), Ghaziabad, Uttar Pradesh 201002 India

**Keywords:** Plant breeding, Plant development

## Abstract

*Dendranthema grandiflora* is an important cut flower with high economic importance in the floriculture industry. Identification of stable and high yielding genotypes of *Dendranthema grandiflora,* hence becomes paramount for ensuring its year-round production. In this context, the genotype by environment interaction effects on 22 chrysanthemum hybrids across six test environments were investigated. The experiment was conducted using Randomized Complete Block Design with three replications for 6 years and data on various agro-morphological and yield-contributing traits were evaluated. Our analysis revealed significant mean sum of squares due to environmental, genotypic and genotype by environment interaction variations for all examined traits. A 2D GGE biplot constructed using first two principal components computed as 59.2% and 23.3% of the differences in genotype by environment interaction for flower yield per plant. The GGE biplot identified two top-performing genotypes, G2 and G5, while the AMMI model highlighted genotypes G17, G15, G6, G5, and G2 as the best performers. Genotype G17 ranked highest for multiple traits, while G2 displayed high mean flower yield as well as stability across all environments. According to AEC line, genotypes G2 and G5 exhibited exceptional stability, whereas genotypes G4, G18 and G19 demonstrated lower stability but maintained high average flower yields. Hence, our findings provide valuable insights into chrysanthemum hybrids that were not only best performing but also hold promise to meet the growers demand of the cut flower industry and can be recommended for large scale commercial cultivation.

## Introduction

The chrysanthemum, scientifically known as *Dendranthema grandiflora* Tzvelev, holds a special place in the world of ornamental horticulture. Commonly known as the ‘Queen of the East’, ‘Autumn Queen’ or ‘Guldaudi’ in India and simply as ‘mum' in America, this perennial plant of the Asteraceae family, is adaptive to north eastern continents, particularly Europe and Asia. "*Chrysanthemum*" word finds its origins in the Greek term "Chryos," signifying gold^9^ and has recently been reclassified as *Chrysanthemum* × *morifolium* (*Dendranthema grandiflorum*). The species name, "*grandiflorum*", underscores its characteristic large flowers. With a basic chromosome number of 9, chrysanthemums can exhibit a wide range of ploidy levels, most commonly as hexaploids^32^. Chrysanthemum's ever-increasing demand in the cut flower industry is attributed to its visually appealing color, extended vase life, sturdy flowers, uniform blooming, tall erect stems, long internodes, normal sprays with prominent center blooms, and the ease with which flower buds open upon reaching their destination. This versatile plant is commercially grown as cut flowers, loose flowers, and potted plants, and the horticultural industry continually seeks new varieties to meet evolving demands^20^. It possesses a significant prominence in the international cut flower trade, ranking second only to the rose among spray chrysanthemums and sixth for disbudded chrysanthemums in the global cut flower market^38^. Notably, it serves as the national flower of Japan, symbolizing optimism and joy. China and Japan lead in chrysanthemum production, with areas of 8475 hectares (2013) and 5230 hectares (2009), respectively. In India, the chrysanthemum cultivation spans over 23.93 thousand hectares area, with production of 470.16 thousand tonnes including 4.54 lakh tonnes of loose flowers and 15.96 thousand tonnes of cut flowers^29^. Beyond its ornamental value, chrysanthemum has found utility in traditional medicine and folk remedies. Its heads are used to make a refreshing tisane believed to enhance vision, soothe sore eyes, alleviate headaches, and combat various ailments such as inflammation, bruises, sprains, snake and centipede bites, rhinitis, diphtheria, cholera, and malaria^34^. Additionally, chrysanthemum has exhibited anti-pyretic and anti-hypertensive properties^36^. The petals have been used in treating fever and wind-heat syndrome^25^, while Chinese cuisine incorporates the flowers in salads, and tea (tisane) is crafted using dried petals^7^. Fully open flowers are harvested in autumn and later dried with a steaming process used in China to reduce bitterness. In Chinese tradition, infusions of chrysanthemum leaves and flowers served medicinal purposes and were even fermented into wine. Overall, it is a versatile plant with both medicinal and ornamental uses.

Chrysanthemum's growth and flowering are significantly influenced by environmental factors, with light and temperature serving as paramount determinants. It's a photosensitive crop, requiring short days for flowering and long days for robust vegetative growth. Precise control of photoperiods enables year-round cultivation^40^. While the plant offers immense potential, the existing cultivars falls short in meeting the demand for new colors, forms, and characteristics. The imperative, therefore, is to identify genotypes suitable for diverse agro-climatic conditions and enhance their stability. The genotypes selected based on correlation between traits can lead to improvements in economically valuable characteristics^18^. To cultivate any species of crop effectively in diverse agro-climatic conditions, the essential factors of its resilience and yield stability must be thoroughly assessed. This evaluation is facilitated by employing a range of statistical tools, involving two key approaches: univariate and multivariate stability statistical analyses^30^. Multivariate techniques, such as cluster analysis, pattern analysis and principal component analysis (PCA) are instrumental in exposing genotype-environment interaction (GEI) patterns^28^. Biplots, a prevalent tool, not only help visualize interaction patterns but also assist in the identification of consistently steady genotypes in various environmental conditions, elucidating the relations between genotypes, environments, and genotype-environment interactions (GEIs)^30^. To study GE interaction, two most commonly used models are the GGE and AMMI biplot. These biplots have been instrumental in conducting varietal stability studies, as seen in multi-location trials, genotype assessment & environmental evaluation^21^. The multiplicative model employed in these analyses integrate two main factors: genotypes (G) and genotype by environment interaction (GE) which becomes valuable in analyzing "which-won-where" pattern in yield data of multi-environment trials, enabling the identification of high yielding cultivars with stability across different test environments^45^.

Researchers have frequently utilized GGE analysis to categorize large scale-environments, assess genotype rankings, and select distinguished and representative environments from the pool of investigations^30^. In contrast, AMMI analysis integrates the examination of primary environmental effects with PCA of GEI. AMMI helps in identifying GEIs within environment characterized by multiple dimensions and representing them on a scatter biplot. While GGE biplots offer a comprehensive view of complex GE interactions in experiments involving breeding lines across multiple environments as well as agronomic examinations^24^. AMMI biplots specifically break down GE interaction effects within the PCA, making it difficult to directly depict genotype effects. The analysis through GGE biplot is widely used statistical tool for creating superior cultivars characterized by phenotypic stability, identifying consistent performing genotypes evaluated among multiple environment conditions, and attaining stability in agricultural yields^19^. Limited research has been conducted on stability analysis in chrysanthemum using methods of GGE and AMMI biplot^42^. Consequently, this present study seeks to recognize hybrids showing consistent yield over a span of six years, employing diverse methodologies of stability analysis. Additionally, the study aims to investigate an impact of environment interactions with the genotypes of *Dendranthema grandiflora* Tzvelev for the flower yield and related components, with an ultimate goal of identifying genotypes with consistent performance and high flower productivity.

## Materials and methods

### Planting material

**Figure 1:**
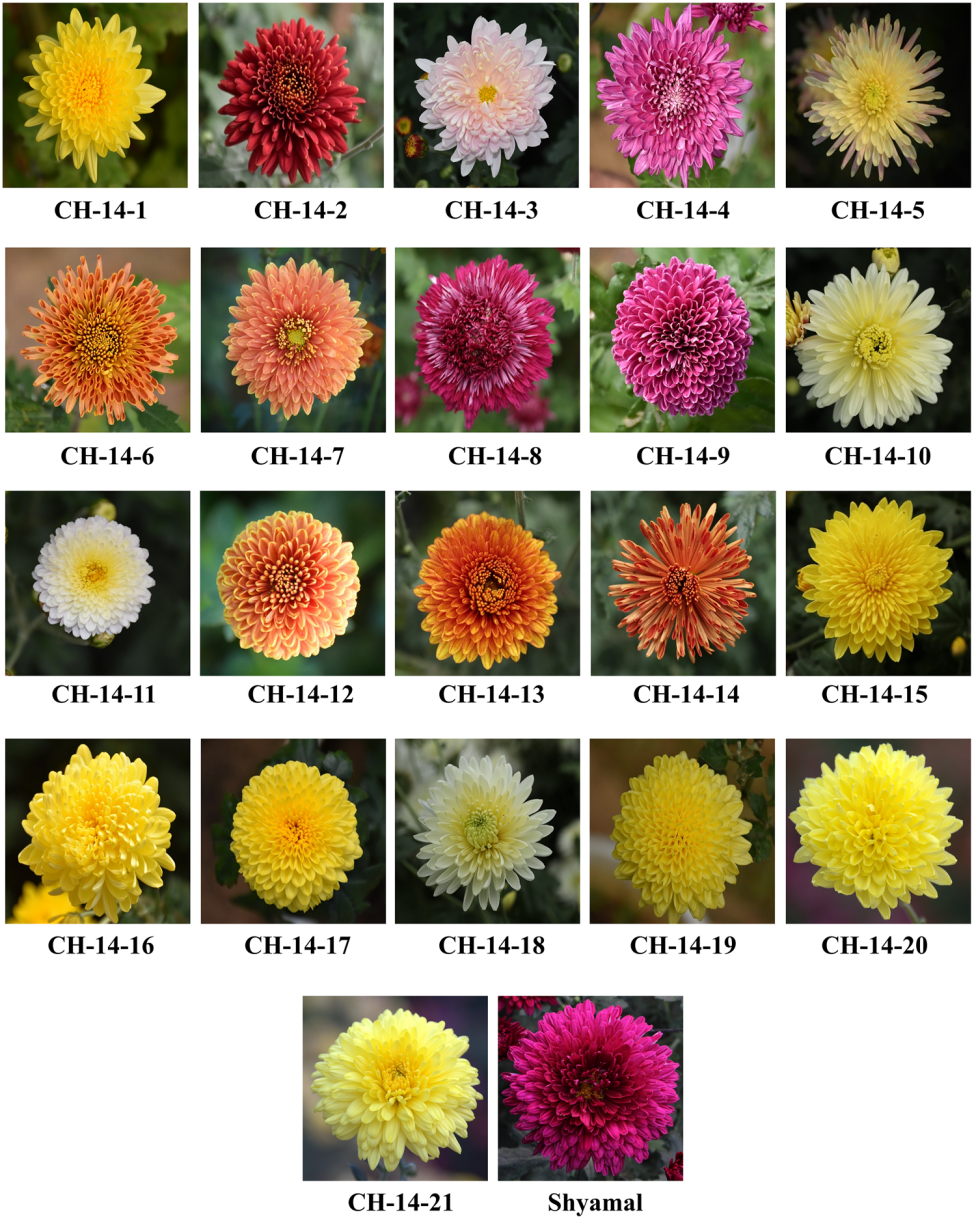
Experimental material used in the investigation.

In this study, twenty-one chrysanthemum genotypes evolved through hybridization method were evaluated along with standard variety ‘Shyamal’ as check for six years. Every year, the genotypes were raised through cuttings (clonal propagation). Shoot tip cuttings of 8–10 cm length were taken from healthy and disease free mother plants. The basal leaves were removed and a cut was given just below the node. The cut end was dipped in a solution of 500 ppm NAA and kept for rooting. The cuttings took 20–25 days for rooting. These rooted cuttings were transplanted in the main field. The list of the material selected is presented in Fig. 1 and along with their source (Parents) in Table 1. The genotypes for the study were obtained from Agrotechnology Division, CSIR-Institute of Himalayan Bioresource Technology, Palampur, Himachal Pradesh, India.

**Table 1:** Pedigree details of the chrysanthemum hybrids used in the study.

Code	Genotype	Pedigree	Code	Genotype	Pedigree
G1	CSIR-IHBT-CH-14–1	YP/WS-5	G12	CSIR-IHBT-CH-14–12	YP/S-6
G2	CSIR-IHBT-CH-14–2	YP/S-19	G13	CSIR-IHBT-CH-14–13	YP/S-52
G3	CSIR-IHBT-CH-14–3	YP/WS-1	G14	CSIR-IHBT-CH-14–14	YP/S-56
G4	CSIR-IHBT-CH-14–4	YP/S-40	G15	CSIR-IHBT-CH-14–15	YP/S-28
G5	CSIR-IHBT-CH-14–5	YP/WS-6	G16	CSIR-IHBT-CH-14–16	YP/WS-4
G6	CSIR-IHBT-CH-14–6	YP/S-23	G17	CSIR-IHBT-CH-14–17	YP/P-3
G7	CSIR-IHBT-CH-14–7	YP/S-22	G18	CSIR-IHBT-CH-14–18	YP/P-2
G8	CSIR-IHBT-CH-14–8	P/S-14	G19	CSIR-IHBT-CH-14–19	YP/WS-3
G9	CSIR-IHBT-CH-14–9	YP/S-14	G20	CSIR-IHBT-CH-14–20	YP/WS-7
G10	CSIR-IHBT-CH-14–10	YP/S-47	G21	CSIR-IHBT-CH-14–21	YP/S-67
G11	CSIR-IHBT-CH-14–11	YP/S-32	G22	Shyamal	

### Experimental site and procedure

The present investigation was carried out under the Council of Scientific and Industrial Research-Institute of Himalayan Bioresource and Technology (CSIR-IHBT), Palampur, H.P., India. The experiment was carried for six cropping seasons from 2015 to 2021 i.e., E1: 2015, E2: 2016, E3: 2017, E4: 2018, E5: 2019 and E6: 2021. The degree of conditions pertaining throughout the seasons varied w.r.t temperature, precipitation, relative humidity and management practices. Experiment was structured in RCBD with 3 replications and 10 plants of each genotype were planted per replication in each cropping seasons. All the cultural practices including pinching, weeding, hoeing, irrigation, staking was carried out as and when required every year. The recommended fertilizer dose of N, P, K (150 kg/ha, 100 kg/ha and100 kg/ha) was also given during final land preparation as well as post-planting. Data was recorded on five random plants per replication for plant height-PH (cm), petiole length-PL (cm), leaf length-LL (cm), leaf width-LW (cm), peduncle thickness-PT (cm), floret length-FL (cm), floret width-FW (cm), flower diameter-FD (cm) and number of flowers per plant-FPP. In addition, international, national, and institutional guidelines were followed at various stages of the experiment.

### Statistical analysis

The AMMI model, integrating analysis of variance with principal components analysis^50^, was employed to estimate the effects of genotype, environment and genotype by environment interaction. The data recorded for various traits of chrysanthemum genotypes was subjected to analysis of variance to identify significant variations among the genotypes, environments and genotype by environment and examining stability among genotypes in different environments. Graphically, stability analysis was done through GGE biplot and AMMI to elucidate the genotype & environment interaction using PBTools v1.3 (International Rice Research Institute, Los Baños, Laguna) software. The GGE Biplot methodology is a combination of two concepts, genotype^12^ and GGE interaction^47^, which facilitate visual examination of the G × E interaction. The GGE and AMMI biplots are graphical representations to illustrate mean and stability-based ranking of G × E interaction and genotypes. These graph formed are based on evaluation across multi-environments (which-won-where), genotype assessment (mean *vs.* stability), and ranking of the environments used for the study based on their distinguished and representative nature, genotype categorization and relationship among environments was allocated for each stability parameter. The following Eq. (1) was used to execute stability analysis through AMMI method^13^:

1 $${\text{Y}}_{{{\text{ge}}}} = \, \mu \, + \, \alpha_{{\text{g}}} + \, \beta_{{\text{e}}} + \Sigma_{{\text{n}}} \lambda_{{\text{n}}} \gamma_{{{\text{gn}}}} \delta_{{{\text{en}}}} + \, \rho_{{{\text{ge}}}}$$

where,

Y_ge_ is yield of the genotype (g) in e environment; µ is grand mean; α_g_ is genotype deviation from µ; β_e_ is deviation from environment; n is single value for IPC_n_ and ${\lambda }_{n}$ is eigenvalue; γ_gn_ is eigenvector for genotype (g) and component (n); δ_en_ is eigenvector for e and n, and ρ_ge_ is residual.

GGE biplot was generated through graphic analysis using single value decomposition as per the Eq. (2) ^47^:

2 $${\text{Y}}_{{{\text{ij}}}} - \, \mu \, - \, \beta_{{\text{j}}} = \, - \xi_{{{\text{i1}}}} \eta_{{{\text{j1}}}} + \, - \xi_{{{\text{i2}}}} \eta_{{{\text{j2}}}} + \, \varepsilon_{{{\text{ij}}}}$$

where,

Y_ij_ is mean of genotype (ith) in environment (jth), µ is genotypes mean, β_j_ is effect of environment jth, ${\lambda }_{1}$ and ${\lambda }_{2}$ are first and second components, respectively, ξ_i1_ and ξ_i2_ are genotypic vectors while, η_j1_ and η_j2_ are first and second components environmental vectors, respectively, and ε_ij_ is residual amount for genotype ith in environment jth.

## Results and discussion

### AMMI analysis of variance for flower yield and its related traits

The result of AMMI analysis of variance for 21 chrysanthemum hybrids along with check variety ‘Shyamal’ and 6 environments is shown in Table 2. This analysis revealed that mean sum of squares due to environments, genotypes and genotypes by environment were significant (*p* ≤ 0.05) for all the traits studied. This implies that there is heterogeneity among the genotypes, test environments over the years. Significantly high differences among different genotypes was mainly due to alterations in environmental factors and genetic composition^19^. The significant sum of square and notable impact of environments during different years lead to variation in environmental means and causing difference in each trait. This variation is useful when examining the genotype by environment interaction effects and assessing the stability of genotypes for expressed traits. The statistically significant interaction effects were observed for the key morphological traits. Similar findings showing significant interaction effects of G × E on flower yield were observed by Shirin et al*.*^37^, indicating need of analysis for cultivar stability.

**Table 2:** AMMI analysis of variance for various traits in chrysanthemum across different environments.

Source of variation	df	PH	Prop	PL	Prop	LL	Prop	LW	Prop	PT	Prop
Environment (E)	5	12,728.89*		2.53*		110.02*		37.60*		0.38*	
Replication	12	13.33		0.10		0.68		0.63		0.002	
Genotype (G)	21	2611.72*		4.59*		32.41*		12.15*		0.03*	
G × E	105	454.42*		0.61*		10.38*		3.37*		0.01*	
PC1	25	1187.15*	62.2	1.98*	77.6	31.82*	73	8.02*	56.7	0.02*	55.4
PC2	23	569.86*	27.5	0.30*	10.7	7.88*	16.6	4.11*	26.7	0.01*	26.6
PC3	21	182.98*	8.1	0.20	6.5	3.42*	6.6	1.54*	9.2	0.005*	10.9
PC4	19	43.55*	1.7	0.14	4.1	1.41	2.5	1.11	6	0.003	5.9
Residuals	252	11.60		0.17		1.34		0.79		0.003	
Total	500	434.00		0.56		7.51		2.71		0.01	

Source of variation	df	FL	Prop	FW	Prop	FD	Prop	FPP	Prop		
Environment (E)	5	10.39*		1.25*		37.05*		7710.95*			
Replication	12	0.09		0.02		0.34		20.07			
Genotype (G)	21	10.83*		1.03*		31.67*		2644.10*			
G × E	105	0.59*		0.40*		1.31*		604.22*			
PC1	25	1.21*	48.6	1.54*	92.2	2.33*	42.1	1446.71*	57		
PC2	23	0.56*	20.7	0.05*	2.7	1.49*	24.8	758.25*	27.5		
PC3	21	0.53*	18	0.04*	2.2	1.03*	15.7	351.69*	11.6		
PC4	19	0.28*	8.5	0.04*	1.7	0.98*	13.5	107.14*	3.2		
Residuals	252	0.07		0.01		0.30		17.44			
Total	500	0.84		0.23		2.41		451.20			

*Significant at *p* ≤ 0.05.

*PH*: Plant height; *PL*: Petiole length; *LL*: Leaf length; *LW*: Leaf width; *PT*: Peduncle thickness; *FL*: Floret length; *FW*: Floret width; *FD*: Flower diameter; *FPP*: Flowers per plant-FPP; *Prop.*: Proportion.

The results also showed significant values for the principal components i.e. PC1 and PC2 for all the traits, however, significant values for PC3 were observed for all traits except PL and PC4 was significant for PH, FL, FW, FD and FPP. Crossa et al. ^8^ proposed that AMMI as a best predictive model involves two, three or four IPCA axes. The cumulative variance was observed above 70% for PC1 and PC2 for each trait. This suggested that the interaction among 22 chrysanthemum hybrids with six environments can be effectively captured by first two components of both genotypes and environments.

### Biplot analysis for explication of multivariate analysis

The primary source of variation in evaluating genotypes under multi-environment trials is the effect of genotype (G) in combination with genotype × environment (G × E) interactions^19^. Biplot analysis helps in effectively capturing the ‘which-won-where’ pattern^47^ to conceptualize the GEI pattern according to the association among genotype and the environment, assessment of stability *versus* mean performance for genotype evaluation across environment and facilitates in the evaluation of representable quality and distinguishing ability in assessing test environment.

#### GGE biplot (‘which‑won‑where’ pattern)

**Figure 2:**
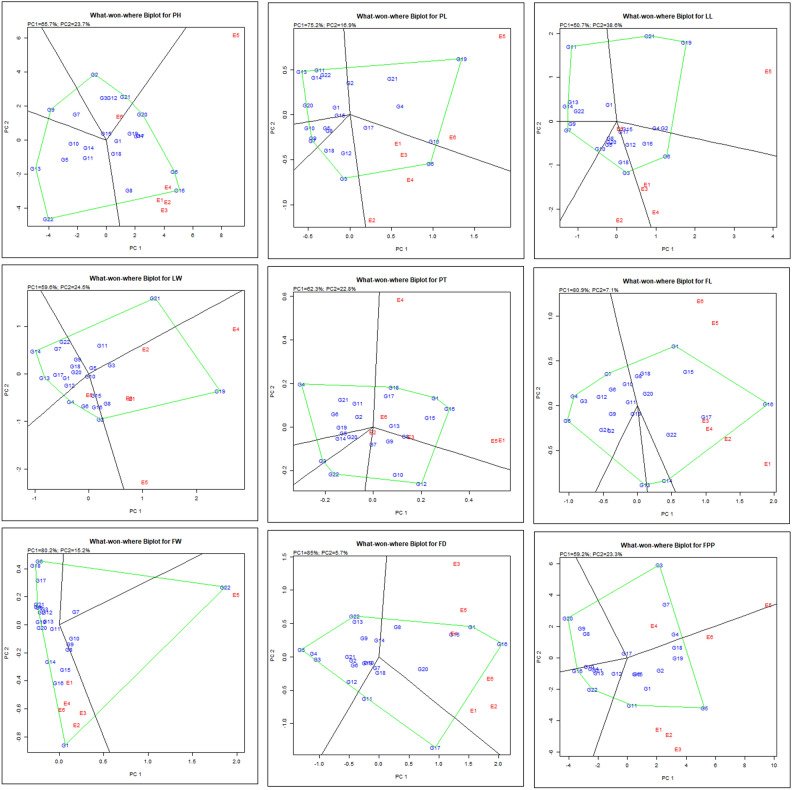
“Which-won-where” biplot pattern depicted in the polygon view of GGE biplot, showcasing main genotype effects plus interaction effect of G × E of 22 chrysanthemum genotypes across test environments.

Assessing chrysanthemum hybrids through which-won-where pattern illustrates that how individually each genotype adapts to a particular environment. The vertex genotypes are connected to form a polygon. The genotypes with most significant vectors in respective directions indicate the degree to which they are reacting to the test environments. All the remaining genotypes fall within polygon and have exhibit smaller vectors, signifies lesser responsiveness in their interaction with environment in that particular sector. Figure 2 illustrated the polygon view of 22 genotypes evaluated under 6 environments for their respective characters. The variation associated with both genotype and genotype-environment (G + G × E) interaction was observed as 89.40%, 92.10%, 89.30%, 84.10% for plant height (PH), petiole length (PL), leaf length (LL), leaf width (LW) and 85.10%, 88.00%, 95.40%, 90.70%, 82.50% for peduncle thickness (PT), floret length (FL), floret width (FW), flower diameter (FD) and yield of flowers per plant (FPP), respectively. The GGE biplot derived from 22 genotypes and 6 environments was divided into 5 (PL, LL & PT), 4 (PH, LW, FL & FPP) and 3 (FW & FD) clockwise fan-shaped sections. The genotype situated at the vertex in each sector represent the maximum value for the respective character in the environment associated with that specific sector. Based on this genotypes G16 followed by G6, G20 were recorded as highly stable and taller in height (PH) in first five environments (E1 to E5) while genotypes G2, G21 performed best in environment E6. For PL, genotypes G19, G10 in E5 & E6 and genotype G6 in E1 to E4 were found stable and best performing. Genotypes G19, G21 in E5 whereas G6 in E1, E3, E4 and G3, G10 in E2 & E6 were found most stable with maximum LL. Genotype G19 performed well for LW in environments (E1 to E5), while it was genotype G2 & G4 in E6. Genotype G16 was observed best for PT followed by G1, G18 in E1, E3 & E6 and whereas it was genotypes G12, G22 in E2. For FL, G1 and G16 exhibited highest performance across the environments (E1 to E6). G22 was recorded as highly stable genotype in E5 for FW, whereas G1, G6, G18 performed well in E1 to E6 except E5. The stability for maximum FD was illustrated by genotypes G1 and G16 in all the environments. However, genotype G3 was identified as both high-yielding and stable in terms of FPP in E4, E5 while genotypes G5, G11 performed well in E1, E2, E3 & E6 environments. Overall, G1 & G16 were the only genotypes found to be best responsive for characters FL and FD in all the environments. While considering the flower yield, genotype G5 and G11 were the best responsive and stable for maximum number of environments.

The studies conducted by Hashim et al.^17^ & Oladosu et al.^30^ in rice depicted that a specific genotype excel across all the tested environments when indicators related to the environment are consolidated in one section while, different genotypes exhibit superior performance when environmental indicators were distributed across different segments of the graph. Hybrid selections situated at vertex of polygon within a biplot sector lacking environmental indicator are consistently underperforming genotypes across test environments^30,39^. Consequently, unveiling ‘which-won-where’ pattern in genotype-environment interaction data matrix is a pivotal characteristic of GGE biplot, extracted by its central components^46^. Similar findings of Bishwas et al.^6^ and Khan et al.^19^ also identified some of the genotypes in wheat & Bambara groundnut, respectively which were adapted specifically to a particular environment under multi-environment trials. Also, Gauch and Zobel^14^ observed vertex genotypes as prevailing genotypes in the corresponding environment.

#### GGE biplot pattern of ‘mean *vs.* stability’ analysis for identification of most stable genotypes

**Figure 3:**
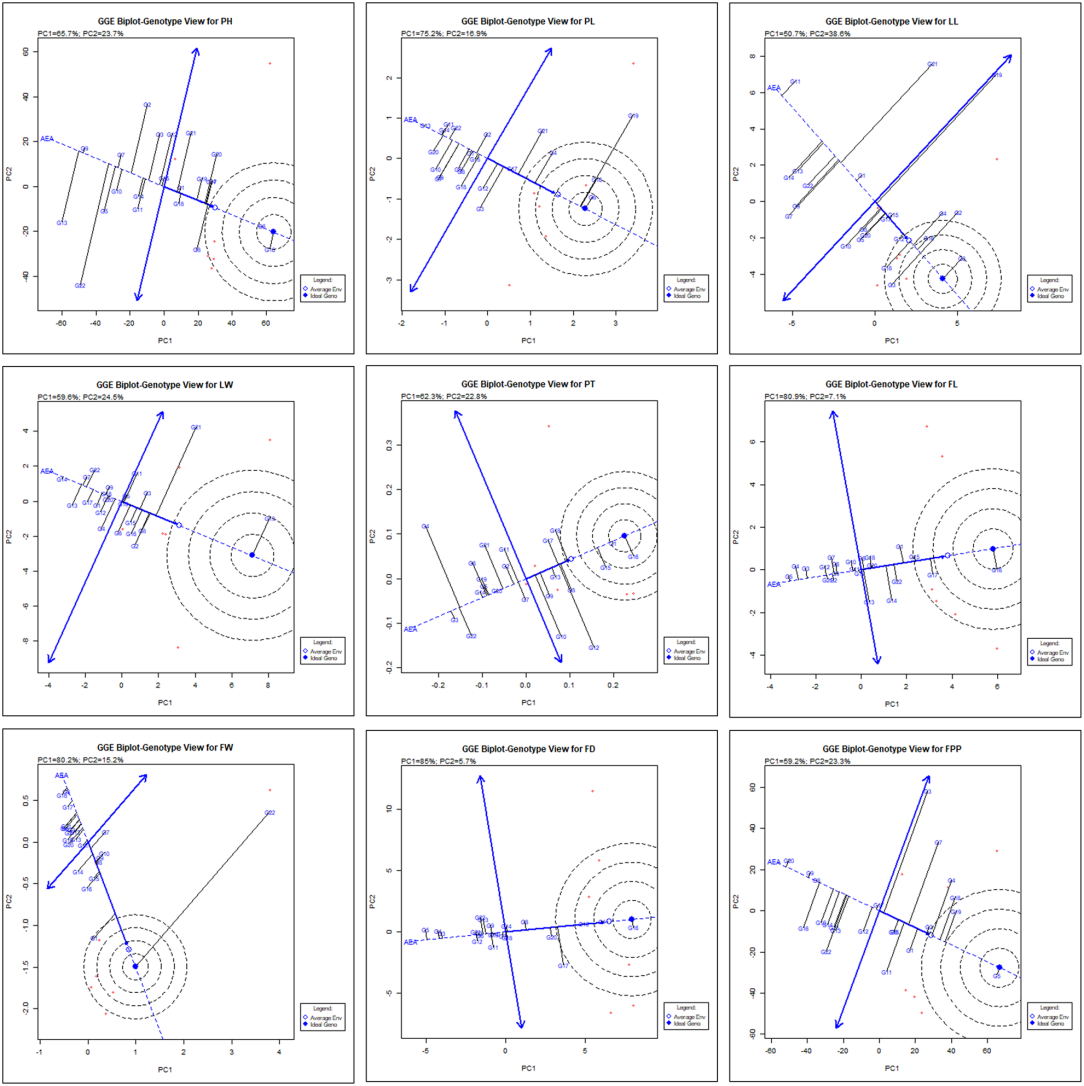
‘Mean *versus* stability’ biplot pattern of 22 chrysanthemum genotypes depicting the interaction effect across test environments for the observed traits.

The ‘mean *vs.* stability’ GGE biplot pattern facilitates simplified assessment of chrysanthemum hybrids based on their average performance and stability. The biplot graph depicted in Fig. 3, comprises of two direct lines viz., the AEC vertical abscissa and AEC horizontal ordinate lines. The genotypes are ranked ascending according to an arrow on the ordinate line, which is connected with greater values of the assessed attributes. The two arrows on a perpendicular line indicate how stable or variable the genotypes are. Any deviation from the ordinate line and biplot origin indicates increased variability and a significant impact from the GE interaction. Therefore, in our investigation, genotype G16 produced highest mean performance for PH followed by G6, G4, G8, G17, G20 and G19 in all the environments. The PH of genotypes was stable for G6, G1, G8 and variable for genotype G22. The highest value for PL was recorded for the genotypes G6, G16, G19 and G4 among all the environments while the most stable were G17 and G6. Genotype G6 was recorded for maximum LL followed by G2, G3, G16, G4, G18 and G12 whereas, genotypes G12, G15 and G17 remarked as highly stable with lower performance for the respective trait. For LW, genotypes G19 followed by G21, G8, G2, G16 and G3 showed highest mean value however, G10, G16, G18 and G20 were most stable but exhibited low performance for the trait. Maximum PT was obtained in genotype G16 followed by G1, G15, G18, G8 and G17 while G1 and G13 were found highly stable among all the environments except E2. In case of FL, genotype G16 followed by G17, G15, G1 and G22 revealed highest mean values but most stable performing was G15, G20, G18 and G6 over the tested environments. The FW was recorded maximum for genotypes G22 and G1 whereas; G8, G9 and G15 were the most stable ones. FD was found maximum in genotype G16 followed by G13 and G17, however, highly stable were G13, G20, G8 and G14. Genotype G5 recorded higher yield in terms of FPP followed by G19, G18, G4, G2 and G1 while G2, G5, G6 and G15 were stable for this trait. Hence, G2 and G5 were the top performing and consistent genotypes for flower yield.

Using mean *vs*. stability biplot graph, stable genotypes were also identified by Hashim et al.^17^, Oladosu et al.^30^, and Sabri et al.^35^ in rice. Genotype positioned near to concentric rings were determined the best performing genotype while their consistency was based on the projection of genotype from AEA abscissa^19^. The genotypes positioned along horizontal axis are regarded as more stable, exhibiting zero projection from the vertical ordinate and vice-versa^30^. The best performing genotypes in the mean *vs*. stability biplot model are those that exhibit the highest AEC prediction (indicating top mean) and the shortest stability vector (indicating maximum stability), as demonstrated by the studies of Farshadfar et al.^11^. Numerous researchers such as Bishwas et al.^6^ in wheat, Ghazvini et al.^15^ in barley, Mostafavi et al.^26^ in rice and Ruswandi et al.^33^ in maize have used this method to find genotypes that perform better and more consistently.

#### ‘Genotype ranking’ for best genotype identification

**Figure 4:**
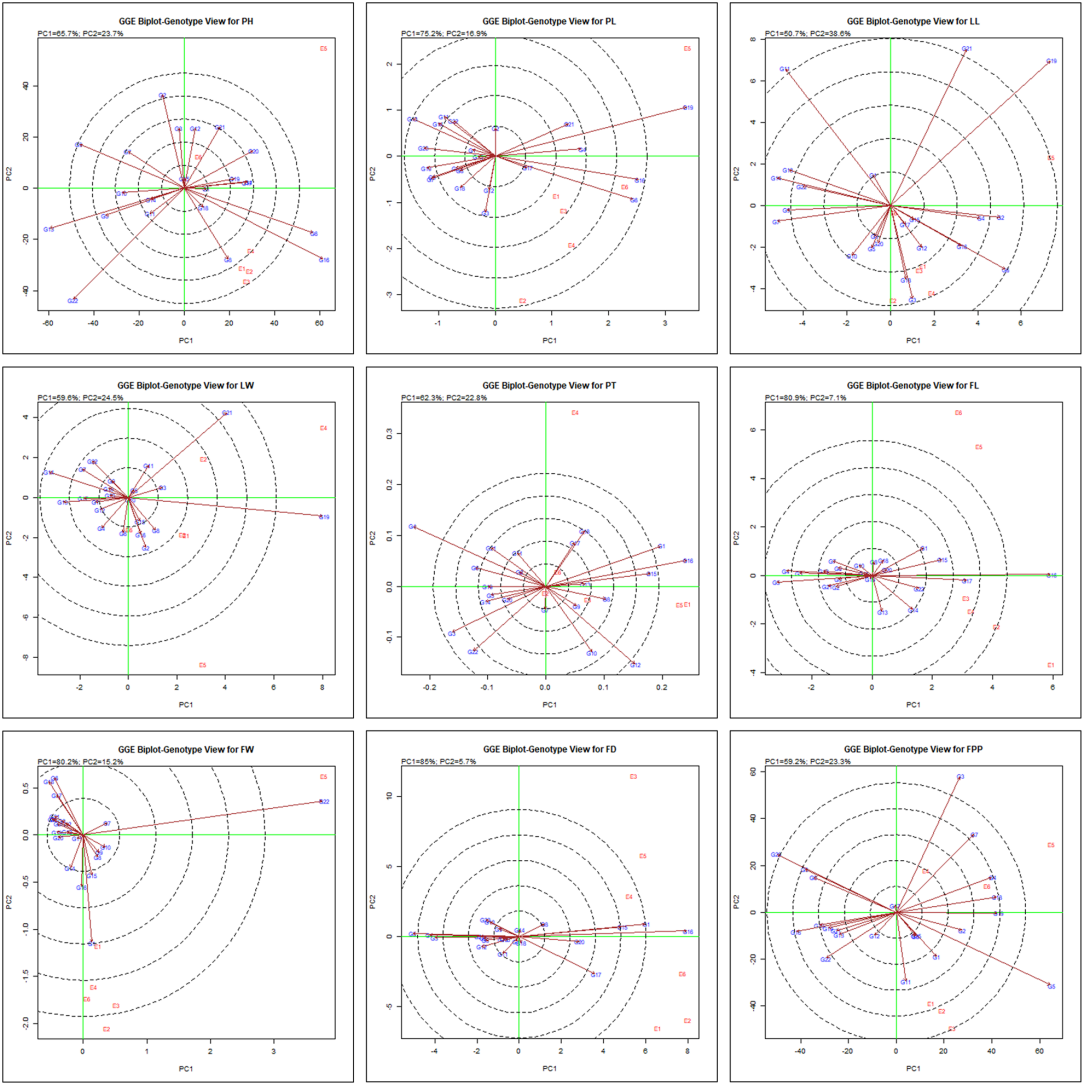
‘Genotypes ranking’ biplot pattern for comparing the genotypes showing interaction effects for genotype and G × E of 22 chrysanthemum genotypes across test environments for the observed traits.

Using genotype ranking pattern depicted in Fig. 4, we can screen the genotypes with optimal performance in comparison to others within a specific environment. Genotypes showing acute angle with all or maximum environments have more than average mean values for different traits were studied. Hence, for PH genotype G1, G4, G6, G16, G19; for PL genotypes G4, G6, G16, G17; for LL genotypes G2, G4, G6, G12, G15, G16, G17; for LW G19; for PT genotypes G1, G8, G13, G15, G16, G17, G18; for FL genotypes G1, G15, G16, G17, G20, G22; for FW genotypes G8, G9, G10; for FD genotypes G1, G8, G15, G16, G20 and for FPP genotypes G2, G4, G5, G19 were regarded as best genotypes based on average mean in all the environments, respectively. Hence, genotypes were ranked as per their best performance for maximum number of traits across the years i.e. G17 > G16 = G15 = G20 > G1 = G20.

The GGE biplot ranking model can pinpoint high-ranking genotypes characterized by significant stability through AEC decisions^39^. The ranking of pyrethrum genotypes belongs to the same family for different traits based on their mean performance and consistency was also examined by Lal et al.^22^. The current research has notably revealed the optimal level of stability and high-performance potential varies between genotypes for specific trait which can be due to the influence of distinct genes governing different traits or result from variation in expression pattern of genes among different genotypes, potentially influenced by diverse environmental factors or various abiotic stresses. A number of prior investigations have documented with comparable findings with only single report on chrysanthemum by Taghipour et al.^42^ and in sunflower belonging to same family^3,44^ and groundnut by Esan et al.^10^.

#### GGE biplot pattern ‘discriminativeness *vs* representativeness’:

**Figure 5:**
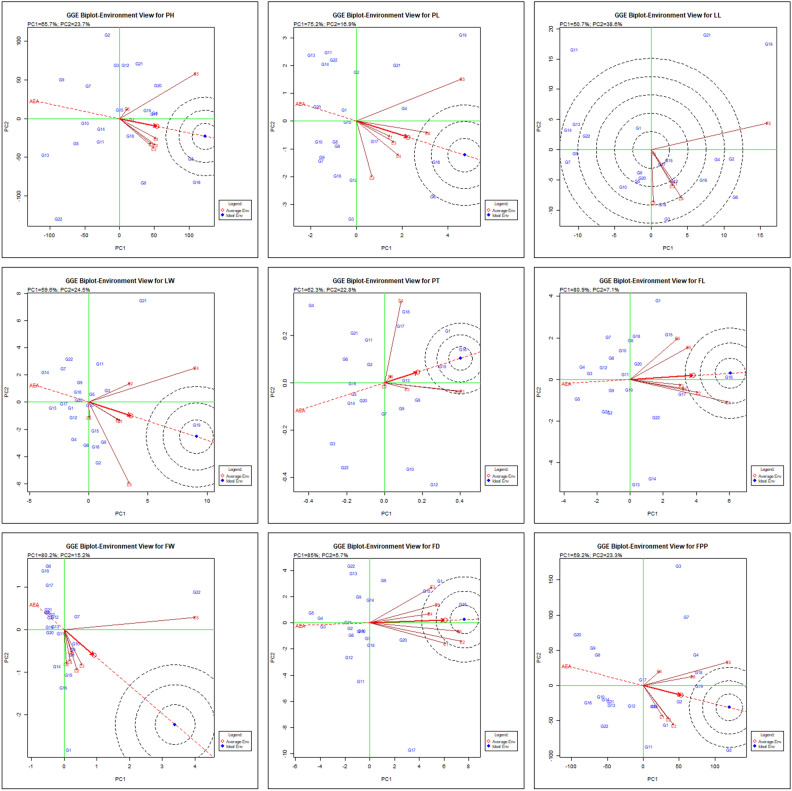
‘Discriminativeness *versus* Representativeness’ biplot pattern for comparing genotypes showing interaction effects for genotype and G × E interaction of 22 chrysanthemum genotypes across test environments for the observed traits.

Discriminativeness refers to the environment’s capability to differentiate between genotypes and representativeness depicts the ability of environment to reflect other assessed environments and constitute fundamental characteristics of a test environment. A small circle designates an ideal environment. Specifically, a best test environment is characterized by most elongated vector (highest discriminating capability) across the test environments and positioned along the AEC abscissa line (most representating)**.** Based on biplot graph (Fig. 5), the environment exhibiting an elongated vector, forming a more acute angle with the abscissa line was E4 for PH, E6 for PL, E1 & E3 for LL and LW; E1 & E5 for PT, E2 for FL, E3 for FW, E6 for FD and FPP depicting a good test environment for respective traits because of their representative as well as discriminating nature for selection of broadly adapted genotypes. Alternatively, environments E5 & E3 for PH, E2 & E5 for PL & LL, E4 & E5 for LW, E4 for PT, E4 & E6 for FL, E5 for FW, E3 & E1 for FD and E5 & E3 for FPP exhibited highest level of discrimination but lack of representativeness.

An ideal environment or locations were also identified based on discriminativeness and representativeness by various workers on different crops including chrysanthemum^42^, safflower^49^ and sunflower^3,44^. According to Yan et al.^47^, the cosine of the angle created by the average environment coordinate or axis (AEC/AEA) and the environment vector is nearly equal to the relationship between the mean genotypic value across environments and the genotypic value inside a particular environment. A smaller angle between the investigated environment axis and vector indicates a more favorable environment than one with larger angles. An arrow indicates the direction of the abscissa line, while a small, concentric circle represents the environment's mean value. An estimate of the test environment ability for discrimination is given by its vector length. According to Oladosu et al.^30^, the size of each environment vector indicates its ability (distinctiveness) to distinguish between genotypes inside that particular environment. Using GGE biplot analysis, distinct environments (E3 and E5) were found to be discriminative and representative for the yield and presence of particular minerals, such as manganese and zinc^4^.

#### Environment ranking or ideal environment assessment:

**Figure 6:**
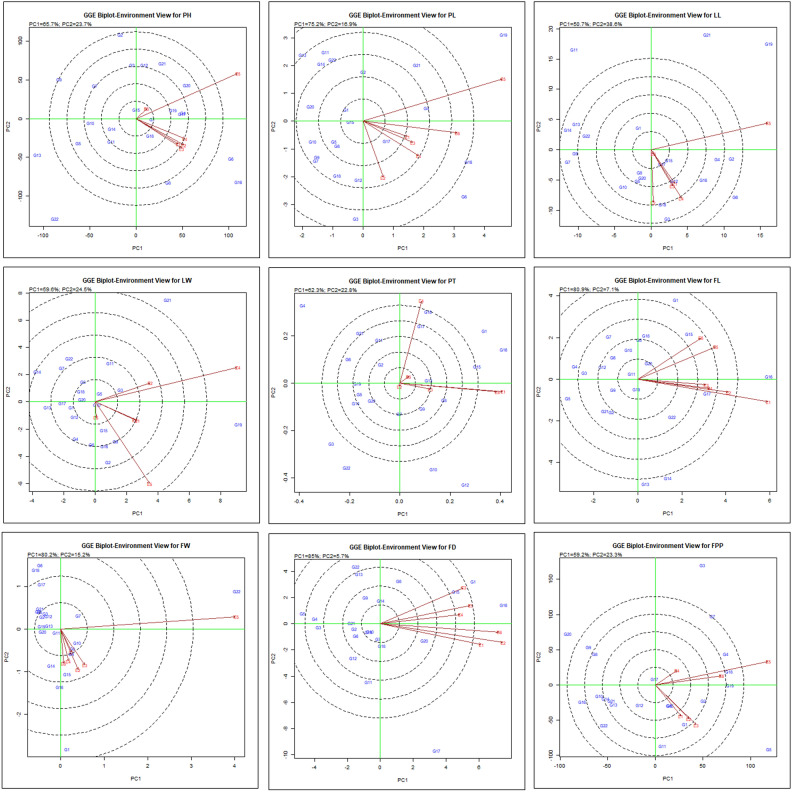
‘Environment Ranking’ biplot pattern for comparing environments depicting interaction effects of genotype and G × E of 22 chrysanthemum genotypes across test environments for the observed traits.

Environment ranking is based on ideal environment in which maximum genotypes performed stable for various traits studied (Fig. 6). According to this, environments are ranked in descending order i.e. for plant height E5 > E6 > E1 = E2 = E3, for petiole length, E2 > E4 > E1 = E3 = E6 > E5, for leaf length, E2 > E3 = E1 = E4 = E6 > E5, for leaf width, E6 > E5 > E1 = E3 > E2 = E4, for peduncle thickness, E1 = E3 = E4 = E5 > E2 > E6, for floret length, E3 = E5 = E6 > E1 = E2 = E4, for floret width, E6 > E1 = E2 = E3 = E4 > E5, for flower diameter, E1 > E2 = E4 = E5 = E6 > E3 and for number of flowers per plant, E1 > E2 = E3 = E5 = E6 > E4 based on the most appropriate environment for the respective traits. Overall, environment E1 was observed to be ideal where maximum genotypes showed stable growth performance for all the traits.

On the basis of biplot graph, evaluation of test environments becomes subsequent crucial step after the identification of multiple environments in determining their discriminative and representative capabilities, correlation and redundancy among environments^19^. Lin and Binns^23^ reported that the impact of environment is significantly shaped by predictable and unpredictable factors on studied genotype. Hashim et al.^17^ described that one environment stands out for selection of genotype based on yield among different environments tested. Ansarifad et al.^3^ also identified best environment on the basis of its distance from concentric circles considered as ideal environment and undesirable which were farthest from the same whereas, Lal et al.^22^ suggested environment II/ year II as a most ideal test environment.

### AMMI model

#### Additive main effects and multiplicative interaction-1 (AMMI 1)

**Figure 7:**
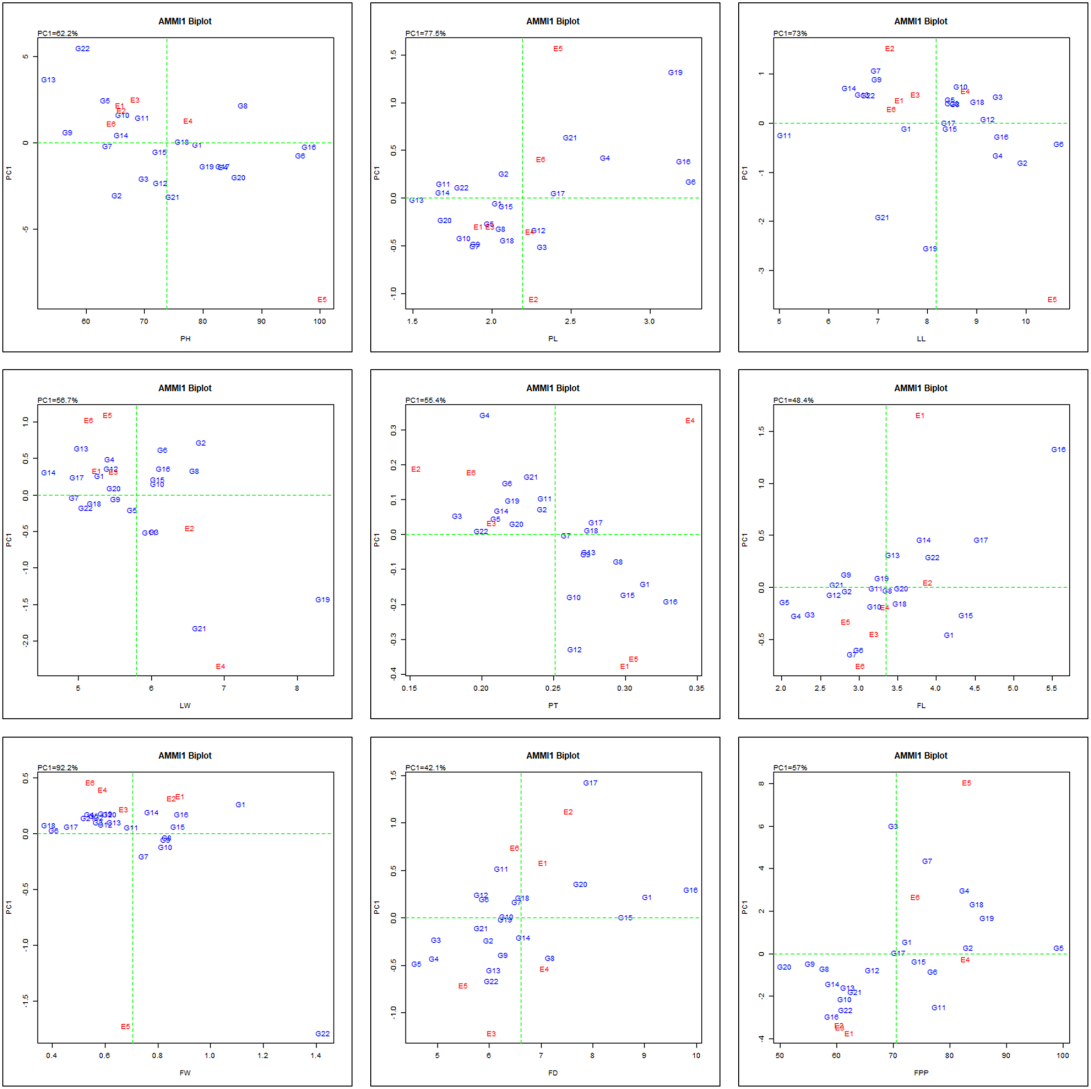
‘AMMI 1’ biplot pattern depicted the main effects of traits and influence of the first principal component (PC1) for both genotype and environment.

The AMMI analysis is a widely accepted and successful technique for selecting elite genotypes in crop species, as evidenced by various studies^1,5,31^. The vertical axis of the biplot in the context of AMMI 1 represents the initial PC1 and the character's major influence, respectively. A PCA1 score near 0 for a genotype or environment indicates a small interaction influence. On the contrary, a positive interaction is shown by a genotype or environment sharing the same sign on the PCA axis, but a negative interaction is shown by a difference in signs. Therefore, for trait plant height (PH), environment E6; E1 & E3 for PL, E6 for LL, E3 for LW, PT & FW, E2 for FL, E1 for FD and E4 for FPP had a negligible first PCA score value i.e. closer to zero than other environments, suggested a diminished interaction effect. This proximity to zero implies superior performance for all genotypes in that particular environment (Fig. 7).

Overall, E3 among all the environments was observed to be acceptable for all the genotypes for maximum number of traits evaluated. Likewise, for PH, the genotypes, G1, G16, G18 and G7; for PL, G1, G13, G14, G15 and G17; for LL, G1, G11, G12, G15, G17; G18, G20, G7 and G9; for LW, G17, G18, G20, G22, G7; for PT, G11, G12, G19, G2, G20, G21 and G8; for FL and FW, genotypes G11, G12, G15, G17, G18, G6, G8 and G9; for FD, genotypes G10, G15 and G19; for FPP, genotypes G17, G2 and G5; attained scores near to zero on PCA1 axis suggested that these genotypes were minimally affected by the environment. Hasan et al.^16^ also differentiated 26 hybrids of rice for stability towards higher grain yield on the basis of their PCA1 scores on AMMI biplot and suggested them to be cultivated preferably as commercial varieties across the studied three locations. Zerehgar et al.^49^ also suggested AMMI biplot as widely used model that simultaneously analyze the genotypes and environments, and therefore utilized it to study stability of 15 genotypes of winter safflower for yield grown for three consecutive cropping seasons.

#### Additive main effects and multiplicative interaction-2 (AMMI 2)

**Figure 8:**
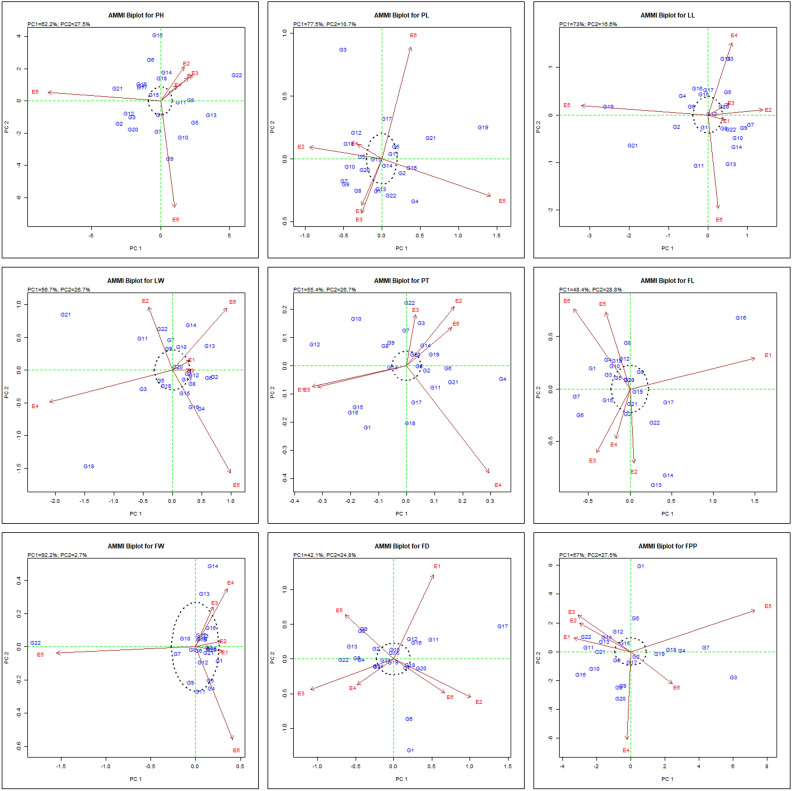
The ‘AMMI 2’ biplot depicting effects of the first two principal components (PC1 and PC2) for genotype alongwith interaction effect of genotype and environment.

AMMI biplot-2 serves as a graphical representation based on principal components (PC1 and PC2) scores and offers insights into genotype stability and interaction effects among genotypes and environments. In Fig. 8, first two principal components of AMMI 2 biplot graph were depicted, explaining 89.7%, 88.2%, 89.6% & 83.4% of variation for PH, PL, LL and LW, respectively. Additionally, it accounted for 82.1%, 77.2%, 94.9%, 66.9% and 84.5% of G + G × E interaction variation for traits, PT, FL, FW, FD and FPP, respectively. Consequently, the distribution of variation indicates that the interaction among 22 tested chrysanthemum genotypes over six years was captured by first two PCs representing genotype & environment. Two lines that connect both vertically and horizontally divide the biplot center (0, 0) into four sections. The distance between the vectors that reflect the environment and genotype and originate from the biplot indicates the degree of interaction, whether the environment influences genotype or vice versa. The genotypes present near to origin that is PC1 equal to zero were more stable than those positioned away from the origin. The high interaction effect was observed in the genotypes positioned farthest from the origin depicting their sensitivity towards environmental interactions. In our study, we found that environment E4 for trait PH, PL, FL and FD; E1 for LL; E1 and E3 for LW; E3 and E6 for PT; E1 and E2 for FW and E6 for FPP had short vector compared to others, indicating a less interactive environment and most suitable for selecting genotype characterized by average performance and adaptability.

The genotypes positioned close to biplot’s origin exhibit consistent responses across all the environments. Therefore, genotypes G15, G1 & G11 for PH; G15, G14, G11, G6, G20 & G5 for PL; G12, G20, G8, G15, G6 for LL; G20, G17, G12, G18, G5, G9, G1 for LW; G5, G13, G20 for PT; G20, G5, G9, G19, G21, G2, G15 for FL; most of the genotypes except G5, G9, G11, G13 & G14 for FW; G3, G7, G10, G14, G15, G18, G19, G21 for FD and G2, G17, G6, G15 for FPP, respectively were near to origin of biplot which indicated their less sensitivity to environment interaction, allowing selection among these genotypes. Our results support the findings of Gauch and Zobel^14^, who concluded that the first two principal components are sufficient to accurately forecast the AMMI model. On the other hand, a few scholars— Sivapalan et al.^41^ and Tariku et al.^43^—have proposed representing multi-environment trials using the first four principal components. Murphy et al.^27^ stated that an environment indicator vector that is shorter and closer to the biplot origin, is less related and can be used as an index to select genotypes with average performance and adaptability. On the other hand, genotypes that exhibit sensitivity to the environmental circumstances that are positioned farther apart and genotypes that cluster close to the biplot's origin imply consistent responses across all tested settings^2^.

### Comparing chrysanthemum genotypes based on mean performance

The mean values for 21 genotypes along with best check Shyamal evaluated over six years is shown in Table 3 (pooled). The values for plant height (PH) ranged from 53.58 cm (G20) to 98.13 cm (G4) with an average value of 73.82 cm. Most of the genotypes except G17 and G20 were found superior for this trait with G4, the tallest in height followed by G14 and G16. Petiole length (PL) varied from 1.52 cm (G20) to 3.26 cm (G14) with a mean value of 2.19 cm. Eight genotypes were superior for this character with G14 having maximum PL followed by G4 and G7 as compared to the check Shyamal. Leaf length (LL) ranged from 5.11 cm (G19) and 10.66 cm (G14) with an average of 8.18 cm. Thirteen genotypes found superior for this trait with highest value for genotype G14 followed by G10 and G4. The trait leaf width (LW) varied from 4.59 cm (G21) to 8.35 cm (G7) with a mean value of 5.80 cm. Nine genotypes showed superiority for this trait with maximum width for G7 followed by G10 and G9. Peduncle thickness varied from 0.18 cm (G11) to 0.33 cm (G4) with average value of 0.25 cm. The superiority compared to the best check was observed for eleven genotypes for this trait with thickest peduncle for G4 genotype followed by G1 and G3. Floret length (FL) and width (FW) varied from 2.04 cm (G13) to 5.59 cm (G4) and 0.39 cm (G6) to 1.42 cm (Shyamal) with average value of 3.35 cm and 0.71 cm, respectively. Only three genotypes were found superior for FL with maximum length for G4 followed by G5 and G3 while none of the genotypes except only the best check for FW. Flower diameter (FD) varied form 4.6 cm (G13) to 9.9 cm (G4) with an average value of 6.62 cm however, the mean value for flower per plant ranged from 51.31 (G8) to 99.78 (G13) with average of 70.52. Eight genotypes for FD were found superior with maximum diameter for G4 followed by G3 and G5 while thirteen for FPP with highest number for G13 followed by G7 and G6. Overall, G3, G4 and G5 were the genotypes which were superior for maximum number of traits.

**Table 3:** Mean performance of chrysanthemum genotypes for flower yield and contributing traits over six years.

Genotypes	PH	PL	LL	LW	PT	FL	FW	FD	FPP
G1	79.05	2.03	7.58	5.29	0.32	4.16	1.11	9.05	73.32
G2	72.67	2.29	9.22	5.45	0.27	2.67	0.60	5.84	66.53
G3	72.58	2.09	8.46	6.08	0.30	4.38	0.88	8.63	73.79
G4	98.13	3.21	9.50	6.17	0.33	5.59	0.89	9.90	58.12
G5	83.34	2.42	8.43	4.98	0.28	4.58	0.47	7.96	70.84
G6	76.47	2.10	9.01	5.22	0.28	3.53	0.39	6.64	83.50
G7	80.63	3.16	8.07	8.35	0.22	3.29	0.60	6.31	86.68
G8	86.11	1.70	8.51	5.48	0.22	3.55	0.62	7.76	51.31
G9	74.83	2.50	7.09	6.66	0.24	2.71	0.54	5.85	61.65
G10	65.21	2.08	9.93	6.68	0.24	2.85	0.58	5.98	83.18
G11	69.85	2.32	9.43	6.03	0.18	2.36	0.57	4.98	69.11
G12	83.39	2.72	9.42	5.43	0.20	2.19	0.54	4.93	82.63
G13	63.26	1.98	8.45	5.74	0.21	2.04	0.56	4.60	99.78
G14	96.58	3.26	10.66	6.15	0.22	2.99	0.41	5.90	77.62
G15	63.58	1.89	6.95	4.94	0.26	2.91	0.75	6.53	76.17
G16	86.85	2.06	8.56	6.59	0.30	3.37	0.84	7.17	57.48
G17	56.76	1.89	6.97	5.51	0.27	2.84	0.83	6.27	55.69
G18	66.28	1.82	8.67	6.08	0.26	3.20	0.83	6.34	63.39
G19	69.56	1.69	5.11	5.97	0.24	3.22	0.70	6.23	78.54
G20	53.58	1.52	6.68	5.05	0.28	3.43	0.63	6.08	61.73
G21	66.01	1.69	6.42	4.59	0.21	3.84	0.78	6.65	59.04
G22	59.42	1.81	6.80	5.10	0.20	3.95	1.42	6.03	61.33
Mean	73.82	2.19	8.18	5.80	0.25	3.35	0.71	6.62	70.52
C.D at 5%	2.49	0.47	1.41	0.91	0.04	0.34	0.07	0.40	2.43
SE (m)	0.87	0.16	0.49	0.32	0.01	0.12	0.03	0.14	0.85
C.V (%)	2.04	12.96	10.42	9.49	9.97	6.06	6.23	3.61	2.09

The primary objective of this work was to assess Chrysanthemum genotypes in order to identify consistent and top performing genotypes across diverse test environments. The substantial sum of square and notable impact of environments indicated that experiments were conducted under varying climatic conditions, leading to differences in environmental means and resulting in variations in flower yield and associated traits. Based on our multivariate analysis, two genotypes, namely G5 (CH-14–5) and G2 (CH-14–2), emerged as highly stable and demonstrated significant potential for high flower yields, making them well-suited for cultivation in all test environments. Conversely, genotypes G4 (CH-14–4), G18 (CH-14–18), G1 (CH-14–1), and G19 (CH-14–19) were identified as suitable for specific environments, displaying lower stability but consistently high flower yields. Additionally, genotypes G6 (CH-14–6) and G15 (CH-14–15) exhibited low flower yields but showcased exceptional stability. These genotypes could be instrumental in breeding programs aimed at enhancing specific phenotypes, given their capacity to quickly adapt to a broad spectrum of environmental conditions while maintaining yield consistency. Our findings underscore the profound influence of environmental factors on flower yield and its component traits, with fluctuations occurring from season to season. The utilization of GGE and AMMI analyses has provided valuable insights into the stability of Chrysanthemum genotypes. Through this comprehensive analysis, we have successfully identified and delineated genotypes that persistently perform well across diverse environments. These well adapted genotypes can be confidently endorsed for commercial cultivation in the sub-temperate Himalayan region, thereby contributing to the sustainable growth of the chrysanthemum industry in this unique and challenging geographic area.

### Supplementary Information

Supplementary Information.

## Data Availability

All data generated or analysed during this study are included in this published article [and its supplementary information file].
