# Author Correction: Stability assessment of selected chrysanthemum (*Dendranthema grandiflora* Tzvelev) hybrids over the years through AMMI and GGE biplot in the mid hills of North-Western Himalayas

**DOI:** 10.1038/s41598-024-67247-8

**Published:** 2024-07-19

**Authors:** Shilpa Kamal, Amit Rana, Rajni Devi, Ravi Kumar, Niketa Yadav, Aniket Anant Chaudhari, Shimran Yadav, Sanatsujat Singh, Bhavya Bhargava, Satbeer Singh, Ramesh Chauhan, Ashok Kumar

**Affiliations:** 1 Agrotechnology Division, Council of Scientific and Industrial Research - Institute of Himalayan Bioresource Technology, Palampur, Himachal Pradesh 176061 India; 2 Academy of Scientific and Innovative Research (AcSIR), Ghaziabad, Uttar Pradesh 201002 India

Correction to: *Scientific Reports* 10.1038/s41598-024-61994-4, published online 19 June 2024

In the original version of this Article, Bhavya Bhargava was omitted as a co-corresponding author. Correspondence and requests for materials should also be addressed to bhavya@ihbt.res.in.

In addition, the Funding section in the original version of this Article was omitted. The Funding section now reads:

“The authors are grateful for the financial support received through HCP 0037 for this study.”

The Acknowledgements section was also incorrect.

“We are grateful to the Director, CSIR-IHBT, Palampur, for his encouragement and providing necessary facilities for this study. The authors are also thankful to Council of Scientific and Industrial Research, New Delhi, India for providing financially support for the study.”

now reads:

“The authors are grateful to the Director, CSIR-IHBT, Palampur, for his encouragement and providing the necessary facilities. CSIR-IHBT communication number is 5593.”

The original Article has been corrected.

