# Supplementary material for: Stability assessment of selected chrysanthemum (Dendranthema grandiflora Tzvelev) hybrids over the years through AMMI and GGE biplot in the mid hills of North-Western Himalayas
Source: Sci Rep. 2024 Jun 19;14:14170. doi: 10.1038/s41598-024-61994-4 (PMC11187203; doi:10.1038/s41598-024-61994-4)
Supplement: Supplementary file 1 — Supplementary Information. [file 41598_2024_61994_MOESM1_ESM.docx]

**Stability assessment of selected chrysanthemum (*Dendranthema grandiflora* Tzvelev) hybrids over the years through AMMI and GGE biplot in the mid hills of North-Western Himalayas**

Shilpa Kamal, Amit Rana, Rajni Devi, Ravi Kumar, Niketa Yadav, Aniket Anant Chaudhari, Shimran Yadav, Sanatsujat Singh, Bhavya Bhargava, Satbeer Singh, Ramesh Chauhan and Ashok Kumar

**Supplementary table 1: Mean performance of chrysanthemum genotypes for plant height, petiole length and leaf length for six years**

| **Genotypes** | **PH** | | | | | | | **PL** | | | | | | | **LL** | | | | | | |
| --- | --- | --- | --- | --- | --- | --- | --- | --- | --- | --- | --- | --- | --- | --- | --- | --- | --- | --- | --- | --- | --- |
|  | **2015** | **2016** | **2017** | **2018** | **2019** | **2021** | **Mean** | **2015** | **2016** | **2017** | **2018** | **2019** | **2021** | **Mean** | **2015** | **2016** | **2017** | **2018** | **2019** | **2021** | **Mean** |
| **CSIR-IHBT-CH-14-1** | 67.98 | 69.83 | 77.99 | 75.84 | 106.40 | 76.28 | **79.05** | 1.79 | 2.17 | 2.07 | 2.01 | 2.27 | 1.90 | **2.03** | 6.65 | 6.37 | 7.67 | 7.44 | 10.23 | 7.10 | **7.58** |
| **CSIR-IHBT-CH-14-2** | 64.43 | 60.33 | 56.98 | 67.69 | 119.97 | 66.62 | **72.67** | 2.12 | 3.00 | 1.89 | 2.25 | 2.03 | 2.47 | **2.29** | 9.25 | 8.83 | 8.19 | 9.69 | 11.37 | 8.02 | **9.22** |
| **CSIR-IHBT-CH-14-3** | 64.59 | 67.63 | 68.37 | 70.30 | 104.37 | 60.24 | **72.58** | 1.82 | 2.37 | 1.92 | 1.98 | 2.23 | 2.20 | **2.09** | 7.96 | 8.37 | 8.41 | 8.66 | 11.53 | 5.81 | **8.46** |
| **CSIR-IHBT-CH-14-4** | 93.03 | 100.70 | 101.19 | 106.10 | 129.30 | 58.45 | **98.13** | 2.77 | 2.83 | 3.02 | 3.16 | 4.07 | 3.43 | **3.21** | 8.80 | 8.73 | 9.57 | 10.04 | 13.10 | 6.74 | **9.50** |
| **CSIR-IHBT-CH-14-5** | 72.92 | 71.96 | 71.70 | 96.63 | 121.77 | 65.07 | **83.34** | 2.00 | 2.40 | 1.98 | 2.66 | 2.63 | 2.83 | **2.42** | 7.59 | 8.03 | 7.47 | 10.07 | 10.97 | 6.44 | **8.43** |
| **CSIR-IHBT-CH-14-6** | 68.53 | 66.96 | 71.31 | 94.03 | 102.30 | 55.66 | **76.47** | 1.85 | 2.57 | 1.93 | 2.53 | 1.60 | 2.10 | **2.10** | 8.80 | 8.40 | 9.16 | 12.09 | 9.93 | 5.68 | **9.01** |
| **CSIR-IHBT-CH-14-7** | 68.69 | 71.52 | 74.02 | 87.93 | 119.93 | 61.67 | **80.63** | 2.34 | 1.70 | 2.52 | 3.01 | 5.33 | 4.07 | **3.16** | 6.00 | 2.53 | 6.48 | 7.69 | 19.37 | 6.32 | **8.07** |
| **CSIR-IHBT-CH-14-8** | 68.38 | 69.89 | 71.08 | 91.25 | 129.30 | 86.79 | **86.11** | 1.46 | 2.07 | 1.51 | 1.94 | 1.67 | 1.57 | **1.70** | 7.40 | 9.00 | 7.69 | 9.87 | 9.63 | 7.45 | **8.51** |
| **CSIR-IHBT-CH-14-9** | 63.35 | 69.14 | 57.89 | 71.74 | 130.10 | 56.77 | **74.83** | 1.95 | 2.23 | 1.79 | 2.21 | 3.83 | 2.97 | **2.50** | 5.32 | 2.80 | 4.87 | 6.03 | 16.03 | 7.47 | **7.09** |
| **CSIR-IHBT-CH-14-10** | 53.95 | 50.27 | 50.89 | 53.98 | 118.90 | 63.24 | **65.21** | 1.79 | 2.13 | 1.69 | 1.80 | 2.87 | 2.17 | **2.08** | 8.80 | 9.40 | 8.30 | 8.81 | 15.50 | 8.77 | **9.93** |
| **CSIR-IHBT-CH-14-11** | 54.23 | 53.98 | 56.39 | 75.70 | 114.03 | 64.76 | **69.85** | 1.85 | 3.00 | 1.91 | 2.58 | 1.43 | 3.13 | **2.32** | 9.20 | 8.67 | 9.57 | 12.90 | 9.87 | 6.37 | **9.43** |
| **CSIR-IHBT-CH-14-12** | 70.03 | 74.43 | 77.59 | 90.11 | 123.07 | 65.13 | **83.39** | 2.22 | 2.40 | 2.46 | 2.86 | 3.83 | 2.53 | **2.72** | 7.88 | 8.03 | 8.75 | 10.13 | 14.33 | 7.40 | **9.42** |
| **CSIR-IHBT-CH-14-13** | 51.23 | 52.87 | 60.79 | 83.91 | 65.57 | 65.16 | **63.26** | 1.64 | 2.00 | 1.94 | 2.68 | 1.70 | 1.93 | **1.98** | 7.13 | 6.93 | 8.46 | 11.68 | 8.80 | 7.70 | **8.45** |
| **CSIR-IHBT-CH-14-14** | 91.07 | 90.43 | 99.87 | 98.89 | 131.83 | 67.42 | **96.58** | 2.85 | 3.13 | 3.13 | 3.11 | 3.70 | 3.63 | **3.26** | 9.85 | 9.20 | 10.80 | 10.68 | 14.60 | 8.84 | **10.66** |
| **CSIR-IHBT-CH-14-15** | 47.80 | 50.09 | 57.68 | 67.72 | 90.67 | 67.56 | **63.58** | 1.60 | 2.54 | 1.93 | 2.26 | 1.47 | 1.53 | **1.89** | 5.74 | 8.33 | 6.92 | 8.12 | 5.60 | 7.01 | **6.95** |
| **CSIR-IHBT-CH-14-16** | 88.11 | 85.24 | 87.29 | 86.17 | 94.37 | 79.92 | **86.85** | 2.11 | 2.40 | 2.08 | 2.05 | 1.83 | 1.87 | **2.06** | 8.93 | 7.50 | 8.81 | 8.73 | 9.27 | 8.16 | **8.56** |
| **CSIR-IHBT-CH-14-17** | 42.87 | 39.23 | 50.91 | 58.00 | 75.53 | 74.02 | **56.76** | 1.70 | 2.37 | 1.99 | 2.30 | 1.43 | 1.57 | **1.89** | 6.06 | 7.10 | 7.17 | 8.21 | 5.97 | 7.30 | **6.97** |
| **CSIR-IHBT-CH-14-18** | 63.14 | 59.91 | 57.81 | 64.28 | 76.97 | 75.61 | **66.28** | 1.82 | 2.40 | 1.67 | 1.86 | 1.43 | 1.73 | **1.82** | 9.24 | 7.73 | 8.48 | 9.43 | 7.93 | 9.23 | **8.67** |
| **CSIR-IHBT-CH-14-19** | 68.23 | 63.74 | 69.96 | 69.84 | 83.20 | 62.37 | **69.56** | 1.55 | 1.37 | 1.59 | 1.59 | 2.00 | 2.07 | **1.69** | 4.42 | 2.47 | 4.53 | 4.52 | 7.77 | 6.94 | **5.11** |
| **CSIR-IHBT-CH-14-20** | 52.46 | 54.75 | 53.10 | 59.98 | 46.20 | 55.00 | **53.58** | 1.49 | 1.50 | 1.51 | 1.40 | 1.73 | 1.50 | **1.52** | 6.87 | 6.47 | 5.90 | 6.00 | 6.70 | 8.13 | **6.68** |
| **CSIR-IHBT-CH-14-21** | 66.94 | 65.26 | 64.80 | 64.33 | 90.17 | 44.55 | **66.01** | 1.40 | 1.67 | 1.66 | 1.55 | 2.00 | 1.87 | **1.69** | 6.27 | 7.57 | 6.70 | 5.23 | 6.30 | 6.43 | **6.42** |
| **Shyamal** | 68.42 | 66.85 | 68.88 | 71.00 | 37.07 | 44.31 | **59.42** | 2.04 | 1.58 | 1.63 | 1.56 | 2.20 | 1.83 | **1.81** | 5.67 | 7.07 | 6.93 | 7.23 | 7.13 | 6.76 | **6.80** |
| **Mean** | **65.93** | **66.14** | **68.48** | **77.52** | **100.50** | **64.39** |  | **1.92** | **2.27** | **1.99** | **2.24** | **2.42** | **2.31** |  | **7.45** | **7.25** | **7.77** | **8.78** | **10.54** | **7.28** |  |
| **C.D._0.05_** | **2.85** | **1.47** | **3.75** | **2.75** | **9.11** | **8.68** |  | **0.50** | **0.91** | **0.55** | **0.65** | **0.64** | **0.75** |  | **1.99** | **1.63** | **2.02** | **2.30** | **2.29** | **0.90** |  |

**Note: PH: Plant height; PL: Petiole length; LL: Leaf length**

**Supplementary Table 2: Mean performance of chrysanthemum genotypes for leaf width, peduncle thickness and floret length for six years**

| **Genotypes** | **LW** | | | | | | | **PT** | | | | | | | **FL** | | | | | | | |  |
| --- | --- | --- | --- | --- | --- | --- | --- | --- | --- | --- | --- | --- | --- | --- | --- | --- | --- | --- | --- | --- | --- | --- | --- |
|  | **2015** | **2016** | **2017** | **2018** | **2019** | **2021** | **Mean** | **2015** | **2016** | **2017** | **2018** | **2019** | **2021** | **Mean** | **2015** | **2016** | **2017** | **2018** | **2019** | **2021** | **Mean** | | |
| **CSIR-IHBT-CH-14-1** | 1.07 | 1.37 | 1.27 | 1.11 | 0.63 | 1.23 | **5.29** | 0.43 | 0.12 | 0.22 | 0.45 | 0.43 | 0.22 | **0.32** | 4.00 | 4.31 | 4.45 | 3.96 | 3.93 | 4.33 | **4.16** |  |  |
| **CSIR-IHBT-CH-14-2** | 0.83 | 0.87 | 0.51 | 0.45 | 0.43 | 0.50 | **5.45** | 0.43 | 0.15 | 0.19 | 0.22 | 0.43 | 0.16 | **0.27** | 3.09 | 3.05 | 2.31 | 2.49 | 2.40 | 2.67 | **2.67** |  |  |
| **CSIR-IHBT-CH-14-3** | 1.11 | 1.27 | 0.82 | 0.69 | 0.73 | 0.63 | **6.08** | 0.43 | 0.18 | 0.19 | 0.40 | 0.43 | 0.17 | **0.30** | 4.19 | 5.13 | 4.29 | 4.52 | 4.60 | 3.57 | **4.38** |  |  |
| **CSIR-IHBT-CH-14-4** | 1.19 | 1.27 | 0.78 | 0.87 | 0.57 | 0.67 | **6.17** | 0.47 | 0.18 | 0.24 | 0.43 | 0.47 | 0.20 | **0.33** | 8.47 | 5.67 | 4.40 | 4.94 | 5.27 | 4.77 | **5.59** |  |  |
| **CSIR-IHBT-CH-14-5** | 0.79 | 0.61 | 0.40 | 0.43 | 0.33 | 0.27 | **4.98** | 0.33 | 0.17 | 0.17 | 0.43 | 0.33 | 0.24 | **0.28** | 5.71 | 5.75 | 3.79 | 4.57 | 3.53 | 4.13 | **4.58** |  |  |
| **CSIR-IHBT-CH-14-6** | 0.55 | 0.49 | 0.41 | 0.37 | 0.23 | 0.27 | **5.22** | 0.33 | 0.11 | 0.25 | 0.47 | 0.33 | 0.16 | **0.28** | 3.90 | 4.12 | 3.21 | 3.13 | 2.67 | 4.13 | **3.53** |  |  |
| **CSIR-IHBT-CH-14-7** | 0.89 | 0.83 | 0.61 | 0.51 | 0.27 | 0.50 | **8.35** | 0.23 | 0.17 | 0.19 | 0.33 | 0.23 | 0.16 | **0.22** | 3.90 | 3.99 | 3.12 | 3.13 | 2.63 | 3.00 | **3.29** |  |  |
| **CSIR-IHBT-CH-14-8** | 0.85 | 0.80 | 0.89 | 0.36 | 0.27 | 0.53 | **5.48** | 0.27 | 0.12 | 0.14 | 0.30 | 0.27 | 0.26 | **0.22** | 4.01 | 4.41 | 2.93 | 3.51 | 2.70 | 3.73 | **3.55** |  |  |
| **CSIR-IHBT-CH-14-9** | 0.74 | 0.75 | 0.48 | 0.50 | 0.27 | 0.47 | **6.66** | 0.23 | 0.14 | 0.16 | 0.40 | 0.23 | 0.25 | **0.24** | 3.13 | 3.48 | 2.51 | 2.79 | 2.07 | 2.27 | **2.71** |  |  |
| **CSIR-IHBT-CH-14-10** | 0.85 | 0.79 | 0.49 | 0.55 | 0.30 | 0.47 | **6.68** | 0.27 | 0.14 | 0.21 | 0.37 | 0.27 | 0.20 | **0.24** | 3.15 | 3.47 | 2.76 | 3.22 | 2.20 | 2.27 | **2.85** |  |  |
| **CSIR-IHBT-CH-14-11** | 0.79 | 0.81 | 0.59 | 0.49 | 0.37 | 0.40 | **6.03** | 0.20 | 0.12 | 0.19 | 0.23 | 0.20 | 0.15 | **0.18** | 2.46 | 2.66 | 2.37 | 2.31 | 2.03 | 2.33 | **2.36** |  |  |
| **CSIR-IHBT-CH-14-12** | 0.78 | 0.69 | 0.44 | 0.46 | 0.23 | 0.63 | **5.43** | 0.13 | 0.20 | 0.17 | 0.43 | 0.13 | 0.15 | **0.20** | 2.29 | 2.25 | 2.02 | 2.31 | 2.03 | 2.20 | **2.19** |  |  |
| **CSIR-IHBT-CH-14-13** | 0.87 | 0.68 | 0.41 | 0.51 | 0.27 | 0.60 | **5.74** | 0.20 | 0.15 | 0.14 | 0.32 | 0.30 | 0.15 | **0.21** | 2.30 | 2.03 | 1.95 | 2.45 | 1.77 | 1.70 | **2.04** |  |  |
| **CSIR-IHBT-CH-14-14** | 0.69 | 0.36 | 0.37 | 0.37 | 0.33 | 0.30 | **6.15** | 0.20 | 0.16 | 0.18 | 0.37 | 0.23 | 0.16 | **0.22** | 2.41 | 3.81 | 3.07 | 3.32 | 2.03 | 3.30 | **2.99** |  |  |
| **CSIR-IHBT-CH-14-15** | 0.90 | 0.81 | 0.73 | 0.46 | 1.07 | 0.50 | **4.94** | 0.30 | 0.19 | 0.25 | 0.30 | 0.30 | 0.22 | **0.26** | 2.20 | 3.86 | 3.01 | 2.73 | 2.73 | 2.93 | **2.91** |  |  |
| **CSIR-IHBT-CH-14-16** | 0.89 | 0.89 | 0.89 | 0.73 | 0.87 | 0.73 | **6.59** | 0.37 | 0.17 | 0.28 | 0.33 | 0.37 | 0.26 | **0.30** | 3.93 | 3.38 | 3.01 | 3.27 | 3.30 | 3.33 | **3.37** |  |  |
| **CSIR-IHBT-CH-14-17** | 1.05 | 0.89 | 0.82 | 0.54 | 0.90 | 0.77 | **5.51** | 0.33 | 0.16 | 0.28 | 0.32 | 0.33 | 0.21 | **0.27** | 3.59 | 3.31 | 2.70 | 2.43 | 2.30 | 2.67 | **2.84** |  |  |
| **CSIR-IHBT-CH-14-18** | 1.02 | 0.95 | 0.76 | 0.69 | 1.00 | 0.57 | **6.08** | 0.37 | 0.19 | 0.26 | 0.23 | 0.37 | 0.17 | **0.26** | 3.41 | 3.31 | 3.15 | 3.23 | 3.17 | 2.93 | **3.20** |  |  |
| **CSIR-IHBT-CH-14-19** | 0.88 | 0.91 | 0.50 | 0.57 | 0.60 | 0.73 | **5.97** | 0.27 | 0.15 | 0.16 | 0.40 | 0.27 | 0.22 | **0.24** | 3.63 | 3.70 | 2.93 | 3.26 | 3.03 | 2.77 | **3.22** |  |  |
| **CSIR-IHBT-CH-14-20** | 0.71 | 0.75 | 0.66 | 0.85 | 0.43 | 0.40 | **5.05** | 0.33 | 0.13 | 0.33 | 0.37 | 0.33 | 0.16 | **0.28** | 4.13 | 4.60 | 3.82 | 3.89 | 2.07 | 2.07 | **3.43** |  |  |
| **CSIR-IHBT-CH-14-21** | 1.04 | 0.96 | 0.96 | 0.91 | 0.40 | 0.40 | **4.59** | 0.23 | 0.13 | 0.20 | 0.30 | 0.23 | 0.19 | **0.21** | 4.86 | 4.88 | 4.26 | 4.10 | 2.30 | 2.60 | **3.84** |  |  |
| **Shyamal** | 0.98 | 1.04 | 1.01 | 0.63 | 4.47 | 0.43 | **5.10** | 0.23 | 0.18 | 0.17 | 0.20 | 0.23 | 0.19 | **0.20** | 4.75 | 4.55 | 4.19 | 3.95 | 3.60 | 2.63 | **3.95** |  |  |
| **Mean** | **0.89** | **0.85** | **0.67** | **0.59** | **0.68** | **0.55** |  | **0.30** | **0.16** | **0.21** | **0.35** | **0.31** | **0.19** |  | **3.80** | **3.90** | **3.19** | **3.34** | **2.83** | **3.02** |  |  |  |
| **C.D._0.05_** | **0.14** | **0.09** | **0.11** | **0.18** | **0.29** | **0.16** |  | **0.13** | **0.05** | **0.04** | **0.10** | **0.13** | **0.05** |  | **1.93** | **0.24** | **0.34** | **0.56** | **0.47** | **0.44** |  |  |  |

**Note: LW: Leaf width; PT: Peduncle thickness; FL: Floret length**

**Supplementary Table 3: Mean performance of chrysanthemum genotypes for floret width, flower diameter and number of flowers per plant for six years**

| **Genotypes** | **FW** | | | | | | | **FD** | | | | | | | **FPP** | | | | | | | | | |  |
| --- | --- | --- | --- | --- | --- | --- | --- | --- | --- | --- | --- | --- | --- | --- | --- | --- | --- | --- | --- | --- | --- | --- | --- | --- | --- |
|  | **2015** | **2016** | **2017** | **2018** | **2019** | **2021** | **Mean** | **2015** | **2016** | **2017** | **2018** | **2019** | **2021** | **Mean** | **2015** | **2016** | **2017** | **2018** | | **2019** | **2021** | | **Mean** | | |
| **CSIR-IHBT-CH-14-1** | 1.07 | 1.37 | 1.27 | 1.11 | 0.63 | 1.23 | **1.11** | 7.75 | 10.91 | 8.84 | 9.74 | 7.03 | 10.03 | **9.05** | 70.93 | 75.27 | 74.33 | 50.52 | 113.67 | | 50.00 | **73.32** | |  |  |
| **CSIR-IHBT-CH-14-2** | 0.83 | 0.87 | 0.51 | 0.45 | 0.43 | 0.50 | **0.60** | 6.75 | 7.11 | 5.15 | 5.50 | 4.90 | 5.63 | **5.84** | 60.13 | 52.07 | 73.93 | 65.11 | 73.67 | | 72.67 | **66.53** | |  |  |
| **CSIR-IHBT-CH-14-3** | 1.11 | 1.27 | 0.82 | 0.69 | 0.73 | 0.63 | **0.88** | 9.25 | 10.17 | 8.59 | 8.37 | 7.57 | 7.83 | **8.63** | 65.00 | 65.80 | 70.60 | 76.66 | 81.33 | | 87.00 | **73.79** | |  |  |
| **CSIR-IHBT-CH-14-4** | 1.19 | 1.27 | 0.78 | 0.87 | 0.57 | 0.67 | **0.89** | 10.57 | 11.07 | 8.71 | 9.92 | 9.13 | 9.97 | **9.90** | 65.33 | 55.27 | 51.20 | 87.35 | 47.00 | | 48.67 | **58.12** | |  |  |
| **CSIR-IHBT-CH-14-5** | 0.79 | 0.61 | 0.40 | 0.43 | 0.33 | 0.27 | **0.47** | 10.00 | 10.47 | 5.57 | 7.55 | 5.93 | 8.20 | **7.96** | 64.73 | 61.87 | 53.13 | 85.18 | 80.33 | | 79.67 | **70.84** | |  |  |
| **CSIR-IHBT-CH-14-6** | 0.55 | 0.49 | 0.41 | 0.37 | 0.23 | 0.27 | **0.39** | 7.20 | 7.64 | 5.77 | 7.31 | 5.10 | 6.80 | **6.64** | 64.40 | 65.20 | 71.80 | 93.26 | 114.67 | | 99.00 | **83.50** | |  |  |
| **CSIR-IHBT-CH-14-7** | 0.89 | 0.83 | 0.61 | 0.51 | 0.27 | 0.50 | **0.60** | 6.67 | 7.15 | 5.43 | 7.34 | 5.13 | 6.13 | **6.31** | 67.87 | 58.60 | 84.53 | 95.50 | 108.33 | | 104.00 | **86.68** | |  |  |
| **CSIR-IHBT-CH-14-8** | 0.85 | 0.80 | 0.89 | 0.36 | 0.27 | 0.53 | **0.62** | 7.96 | 8.74 | 6.24 | 8.45 | 6.67 | 8.50 | **7.76** | 38.18 | 36.00 | 37.05 | 90.27 | 51.00 | | 52.00 | **51.31** | |  |  |
| **CSIR-IHBT-CH-14-9** | 0.74 | 0.75 | 0.48 | 0.50 | 0.27 | 0.47 | **0.54** | 6.15 | 6.41 | 5.41 | 6.25 | 4.87 | 6.00 | **5.85** | 60.87 | 64.47 | 56.27 | 84.02 | 67.00 | | 46.67 | **61.65** | |  |  |
| **CSIR-IHBT-CH-14-10** | 0.85 | 0.79 | 0.49 | 0.55 | 0.30 | 0.47 | **0.58** | 6.73 | 6.55 | 5.14 | 7.75 | 4.67 | 5.07 | **5.98** | 71.00 | 69.80 | 75.20 | 96.29 | 95.67 | | 91.33 | **83.18** | |  |  |
| **CSIR-IHBT-CH-14-11** | 0.79 | 0.81 | 0.59 | 0.49 | 0.37 | 0.40 | **0.57** | 5.34 | 6.31 | 5.05 | 5.60 | 3.67 | 3.90 | **4.98** | 36.33 | 33.60 | 37.60 | 90.67 | 124.00 | | 98.00 | **69.11** | |  |  |
| **CSIR-IHBT-CH-14-12** | 0.78 | 0.69 | 0.44 | 0.46 | 0.23 | 0.63 | **0.54** | 5.23 | 5.31 | 4.47 | 6.54 | 3.83 | 4.20 | **4.93** | 61.68 | 66.33 | 60.47 | 93.16 | 119.33 | | 94.00 | **82.63** | |  |  |
| **CSIR-IHBT-CH-14-13** | 0.87 | 0.68 | 0.41 | 0.51 | 0.27 | 0.60 | **0.56** | 4.67 | 4.91 | 4.17 | 5.86 | 3.93 | 4.03 | **4.60** | 91.13 | 94.53 | 95.13 | 97.07 | 122.33 | | 95.00 | **99.78** | |  |  |
| **CSIR-IHBT-CH-14-14** | 0.69 | 0.36 | 0.37 | 0.37 | 0.33 | 0.30 | **0.41** | 5.17 | 7.75 | 5.48 | 6.47 | 4.27 | 6.27 | **5.90** | 68.69 | 74.67 | 64.40 | 93.09 | 80.67 | | 80.00 | **77.62** | |  |  |
| **CSIR-IHBT-CH-14-15** | 0.90 | 0.81 | 0.73 | 0.46 | 1.07 | 0.50 | **0.75** | 7.10 | 7.65 | 6.11 | 6.78 | 4.90 | 6.60 | **6.53** | 60.02 | 59.53 | 38.00 | 90.13 | 130.67 | | 77.33 | **76.17** | |  |  |
| **CSIR-IHBT-CH-14-16** | 0.89 | 0.89 | 0.89 | 0.73 | 0.87 | 0.73 | **0.84** | 7.77 | 7.15 | 6.82 | 7.53 | 6.87 | 6.90 | **7.17** | 53.54 | 46.80 | 38.00 | 87.15 | 58.00 | | 63.33 | **57.48** | |  |  |
| **CSIR-IHBT-CH-14-17** | 1.05 | 0.89 | 0.82 | 0.54 | 0.90 | 0.77 | **0.83** | 6.87 | 6.42 | 5.86 | 6.57 | 6.03 | 5.83 | **6.27** | 48.41 | 41.60 | 38.33 | 83.26 | 56.67 | | 63.00 | **55.69** | |  |  |
| **CSIR-IHBT-CH-14-18** | 1.02 | 0.95 | 0.76 | 0.69 | 1.00 | 0.57 | **0.83** | 7.18 | 7.09 | 6.00 | 6.59 | 4.97 | 6.20 | **6.34** | 59.11 | 54.13 | 58.67 | 87.08 | 56.00 | | 53.33 | **63.39** | |  |  |
| **CSIR-IHBT-CH-14-19** | 0.88 | 0.91 | 0.50 | 0.57 | 0.60 | 0.73 | **0.70** | 7.13 | 6.99 | 4.56 | 6.31 | 5.27 | 7.13 | **6.23** | 79.74 | 76.53 | 77.60 | 85.47 | 69.33 | | 79.67 | **78.54** | |  |  |
| **CSIR-IHBT-CH-14-20** | 0.71 | 0.75 | 0.66 | 0.85 | 0.43 | 0.40 | **0.63** | 6.15 | 6.33 | 6.26 | 5.96 | 6.00 | 5.80 | **6.08** | 59.84 | 58.18 | 60.12 | 66.24 | 61.67 | | 65.33 | **61.73** | |  |  |
| **CSIR-IHBT-CH-14-21** | 1.04 | 0.96 | 0.96 | 0.91 | 0.40 | 0.40 | **0.78** | 7.24 | 7.34 | 7.05 | 6.86 | 5.00 | 6.43 | **6.65** | 57.20 | 57.33 | 55.90 | 63.03 | 62.33 | | 59.33 | **59.04** | |  |  |
| **Shyamal** | 0.98 | 1.04 | 1.01 | 0.63 | 4.47 | 0.43 | **1.42** | 6.12 | 6.25 | 6.55 | 6.49 | 5.33 | 5.47 | **6.03** | 64.94 | 63.89 | 61.13 | 60.84 | 52.00 | | 66.33 | **61.33** | |  |  |
| **Mean** | **0.89** | **0.85** | **0.67** | **0.59** | **0.68** | **0.55** |  | **7.05** | **7.53** | **6.06** | **7.08** | **5.50** | **6.50** |  | **62.23** | **60.52** | **60.61** | **82.79** | **82.99** | | **73.89** |  | |  |  |
| **C.D._0.05_** | **0.14** | **0.09** | **0.11** | **0.18** | **0.29** | **0.16** |  | **0.49** | **1.68** | **0.52** | **0.56** | **0.83** | **0.77** |  | **4.76** | **4.59** | **7.35** | **3.00** | **8.33** | | **10.48** |  | |  |  |

**Note: FW: Floret width; FD: Flower diameter; FPP: Flowers per plant**
